# Large sliding hiatus hernia: incidental finding in myocardial perfusion scintigraphy performed with SPECT/CT technique

**DOI:** 10.1007/s12350-020-02265-3

**Published:** 2020-07-21

**Authors:** Katarzyna Jóźwik-Plebanek, Marek Cacko, Jacek Wnuk, Anna Teresińska

**Affiliations:** Department of Nuclear Medicine, National Institute of Cardiology, Alpejska 42, 04-628 Warsaw, Poland

Myocardial perfusion scintigraphy (MPS) is a widely used noninvasive cardiac imaging test in the diagnosis of coronary artery disease (CAD), with high sensitivity and specificity for CAD.^1^ Low-dose computed tomography (CT) performed along with MPS for the purposes of attenuation correction (AC) of scintigraphic data has mainly auxiliary function but the incidence of noncardiac findings reaches almost 2%.^2^

**Figure 1:**
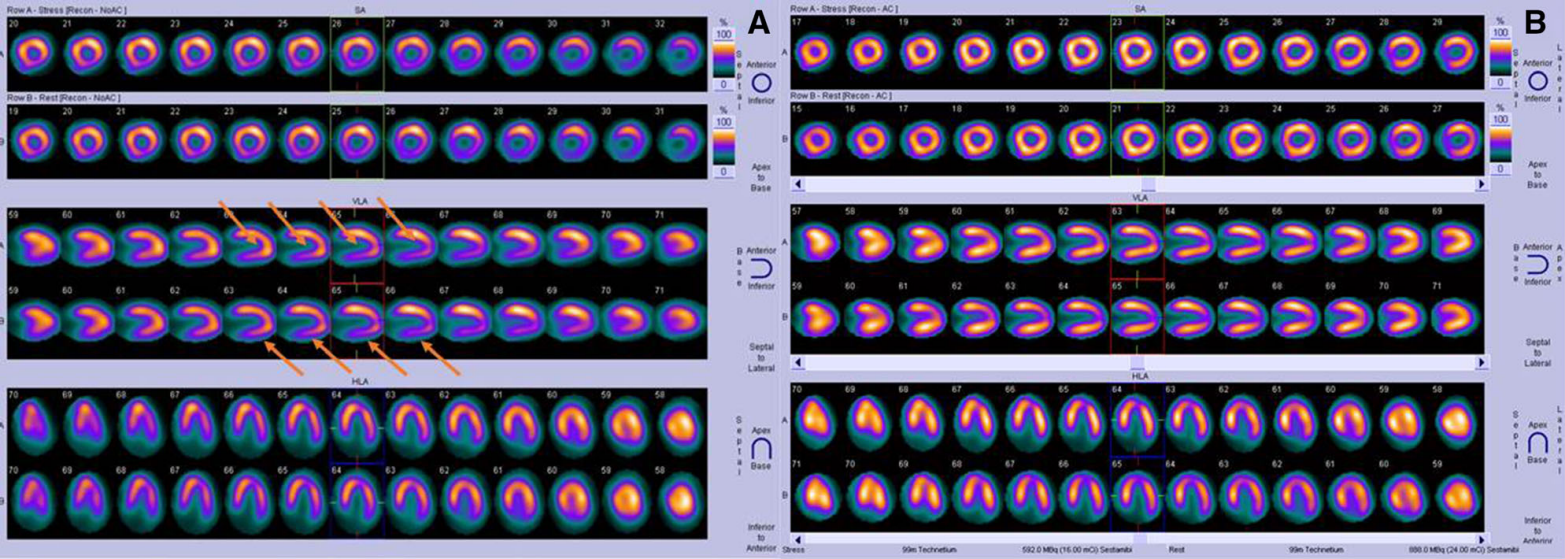
In MIBI SPECT/CT no reversible perfusion defects were found. Moderate fixed perfusion defects in inferior wall of left ventricle were seen in images without attenuation correction (red arrows). After correction of attenuation—the inferior wall perfusion was normalized. (**A**) Myocardial perfusion MIBI SPECT/CT—study without attenuation correction. (**B**) Myocardial perfusion MIBI SPECT/CT—study with attenuation correction

**Figure 2:**
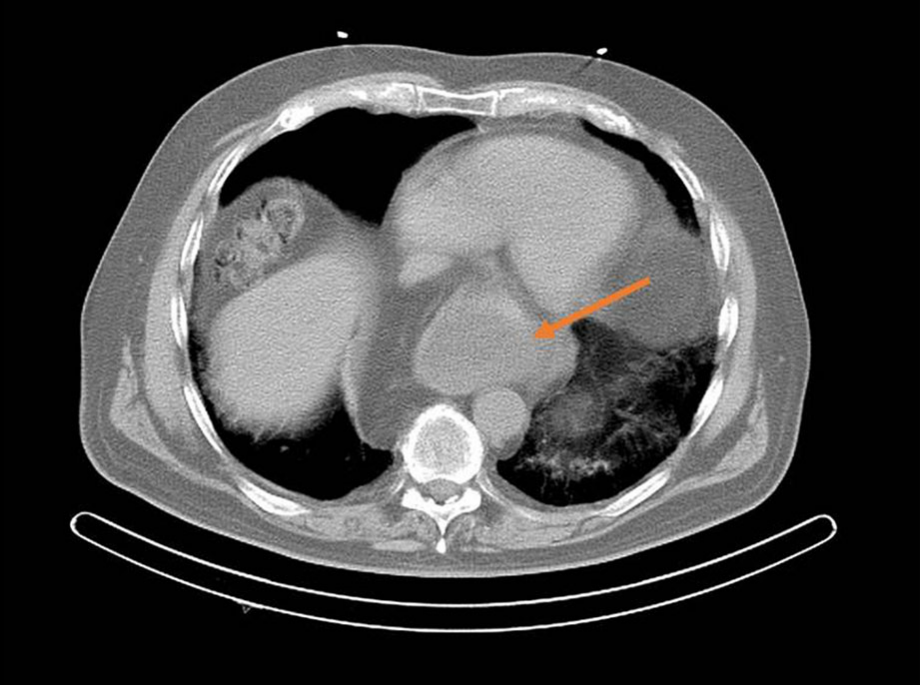
Image of computed tomography made for the purposes of attenuation correction during SPECT/CT. Suspicion of large sliding hernia of the diaphragm was raised (red arrow)

**Figure 3:**
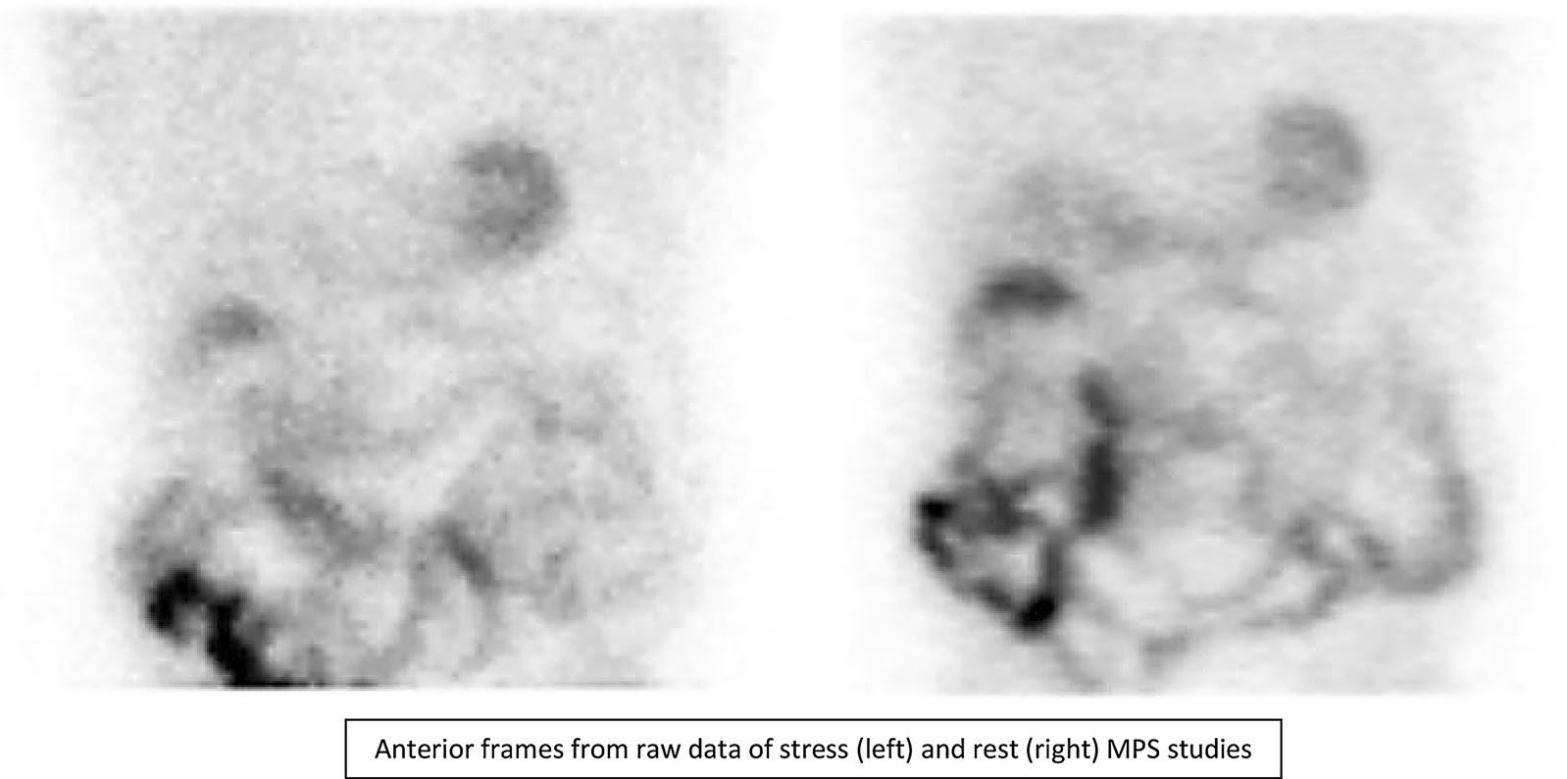
Anterior frames from raw data of stress (left) and rest (right) MPS studies

**Figure 4:**
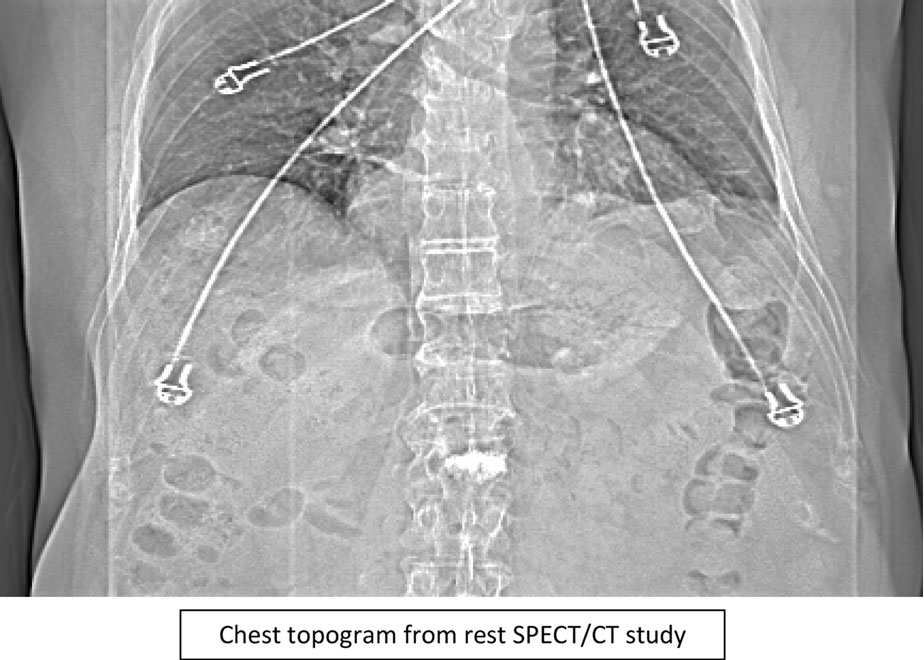
Chest topogram from rest SPECT/CT study

**Figure 5:**
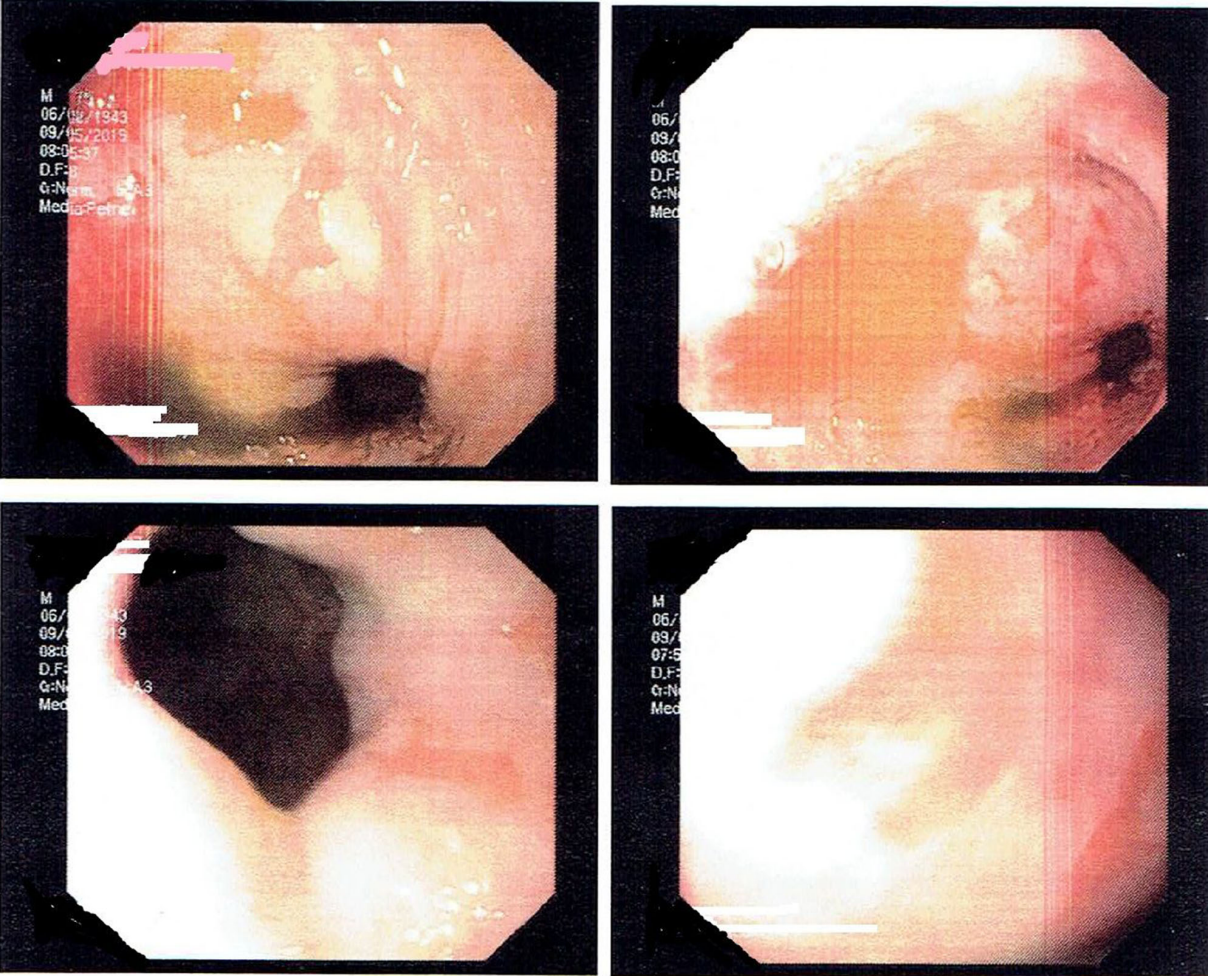
Sliding hiatus hernia seen on gastroscopy. Sliding hiatal hernia is an independent factor for gastroesophageal reflux disease—a prevalent entity which affects quality of life, and if not treated may lead to serious complications including Barrett esophagus—a precursor to esophageal adenocarcinoma.^3^ Nonspecific symptoms including chest pain may lead to false suspicion of coronary artery disease in the patient

**Figure 6:**
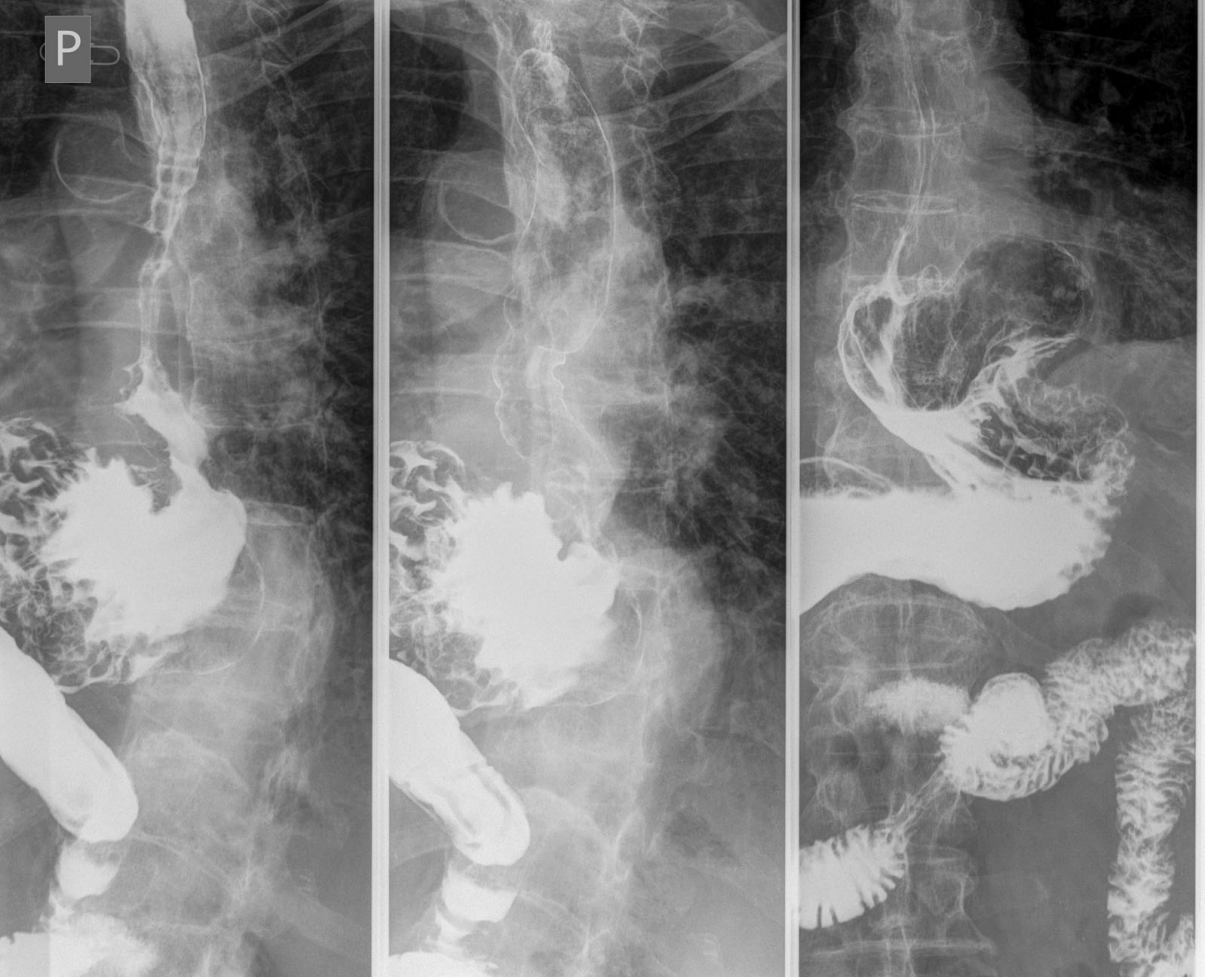
Features of sliding hiatus hernia in x-ray barium study

A 76-year-old patient with history of hypertension, diabetes mellitus, and hypercholesterolemia was referred to nuclear medicine department because of recurrent, unspecific chest pain, appearing for several years. MPS study was performed with [^99m^Tc]Tc-methoxyisobutylisonitrile (MIBI) single-photon emission computed tomography (SPECT)— no reversible perfusion defects corresponding to ischemia were found. In CT carried out for the purposes of AC, a suspicion of large hiatus hernia was found (Figures 1, 2, 3, and 4). In gastroscopy and x-ray barium study performed afterwards, a large sliding hiatus hernia was confirmed (Figures 5, 6). Intestinal metaplasia in the esophagus with pathomorphological suspicion of Barrett’s esophagus and mild chronic gastritis was observed in gastroscopy additionally. After the patient was treated with esmoprazole, a resolution of all the symptoms was observed.

In this case, MPS SPECT/CT conducted with careful analysis of low-dose CT images helped to diagnose the proper cause of symptoms and to protect the patient from severe complications and improper treatment. Presented case shows the importance of analyzing the whole available data collected during a scintigraphy study, including low-dose CT images which can suggest anatomical abnormalities to be confirmed by dedicated methods.
